# Advancements in the clinical application of gene methylation for early cancer detection

**DOI:** 10.3389/freae.2024.1430294

**Published:** 2024-07-19

**Authors:** Xin Xu, Yanru Guo, Mulin Liu, Yunxiang Hu, Shijun Li

**Affiliations:** ^1^ Department of Clinical Laboratory, The First Affiliated Hospital of Dalian Medical University, Dalian, Liaoning, China; ^2^ College of Laboratory Medicine, Dalian Medical University, Dalian, Liaoning, China; ^3^ Department of Laparoscopic Surgery, The First Affiliated Hospital of Dalian Medical University, Dalian, China

**Keywords:** cancer, gene methylation, biomarkers, methylation detection methods, epigenetics

## Abstract

This review provides an overview of common assays used to screen for gene methylation and early biomarkers of methylation in various cancers. DNA methylation, one of the most well-studied epigenetic modifications, plays a crucial role in normal cell and tissue development. It is increasingly utilized as a biomarker for early cancer and precancerous lesion detection. In this review, we describe common methods associated with gene methylation, including bisulfite sequencing PCR (BSP), pyrosequencing technology (PYR), methylation-specific polymerase chain reaction (MS-PCR/MSP), methylation-sensitive high-resolution melting (MS-HRM), methylation sensitive single nucleotide primer extension (MS-SnuPE), Epityper, Droplet digital PCR (ddPCR), methylation-sensitive restriction enzyme (MSRE) analysis, COBRA and PacBio SMRT sequencing. Additionally, we summarize methylation markers and their sample types for early cancer screening, focusing on colorectal cancer, hepatocellular carcinoma, gastric cancer, pancreatic cancer, esophageal cancer (digestive system), lung cancer (respiratory system), breast cancer, ovarian cancer, cervical cancer (female reproductive system), bladder cancer, and prostate cancer (urinary system). Furthermore, we discuss the recent detection of methylation biomarkers in clinical samples such as blood, urine, sputum, feces, and tissues. The aim of this review is to summarize early methylation biomarkers that are expected or have already been clinically applied. For future large-scale studies or the integration of available methylome level data, the discovery of sufficiently sensitive clinical biomarkers is essential.

## 1 Introduction

DNA methylation is a crucial epigenetic regulatory mechanism that plays a pivotal role in tumor development. The human genome contains approximately 2,80,000 CpG sites (cytosine-phosphate-guanine sites). These CpG sites exhibit different distributions across the genome, and CpG dinucleotide clusters are found at expected frequencies, forming regions known as “CpG islands” (CGI) (Gardiner-Garden and Frommer, 1987). In mammalian cells, DNA is methylated at approximately 70%–80% of CpG cytosine C5 sites, but also at non-CpG sites (He and Ecker, 2015). Specific DNA methylation at CpG and non-CpG sites is essential for chromatin regulation. The DNA methyltransferase DNMT3A interacts with its TRD and RD loops with target sites surrounded by variable DNA sequences. DNA methylation typically occurs at CpG sites, where cytosine in the CpG dinucleotide environment is methylated at the fifth carbon position by DNA methyltransferases (DNMTs), resulting in 5-methylcytosine (5 mC) (Bestor, 1988) (Figure 1). During tumor development, normal epigenetic processes are disrupted, leading to global changes in DNA methylation patterns (Jones and Baylin, 2007). Cancer cells are characterized by genome-wide hypomethylation, but tumor suppressor initiators may contain associated CpG islands and therefore can become hypermethylated before or during tumorigenesis (Baylin and Jones, 2011). Hypermethylation of promoter CpG islands is a common phenomenon and represents an alternative mechanism for inactivating tumor suppressor genes, DNA repair genes, cell cycle regulators, and transcription factors.

**FIGURE 1:**
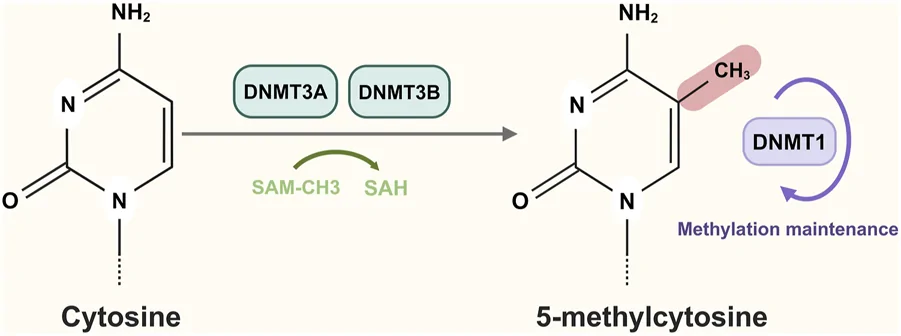
DNA methylation occurs at CpG sites and cytosine is methylated by DNA methyltransferases (DNMT3A and DNMT3B) to generate 5-methylcytosine. Figure created with BioRender.com.

Accurate early diagnosis and prediction of cancer cell metastasis are crucial for effective treatment. Biomarkers play a significant role in aiding clinicians in determining tumor classification, degree of progression, and molecular dysregulation mechanisms. *MLH1* promoter CpG sites have been found to exhibit high specificity in detecting *MLH1* methylation, making them suitable for screening Lynch syndrome (LS) (Tan et al., 2024). Recent studies (Butler et al., 2020; Della Monica et al., 2022; Leske et al., 2023) have demonstrated that methylation of the O6-methylguanine-DNA methyltransferase (*MGMT*) promoter can help evaluate prognosis and predict drug response in glioblastoma patients. Moreover, nanopore sequencing has been identified as an effective method for detecting the methylation status of the *MGMT* promoter (Halldorsson et al., 2024). Hypermethylated *BCAT1*, *IKZF1*, and *SEPT9* are found to be differentially methylated in both colorectal and gastroesophageal adenocarcinomas. The detection of methylated circulating tumor DNA (ctDNA) of *SEPT9* in blood is used as a screening method for colorectal cancer (CRC) (Laven-Law et al., 2024). Early screening of methylation markers and cancer type diagnosis through specific methods can greatly improve the effectiveness of existing cancer treatments and enhance patient recovery rates. Methylation biomarkers are not only useful for diagnosing and staging patients but also for subsequent monitoring and assessing patient prognosis. Therefore, the examination of DNA methylation profiles is increasingly recognized as an important biomarker for tumor diagnosis, prognosis, treatment response, and disease surveillance (Heyn and Esteller, 2012). DNA methylation biomarkers are also considered valuable noninvasive diagnostic tools for cancer detection.

Liquid biopsy (LB) is a less invasive method that reduces the need for tissue biopsies. However, LB faces challenges in clinical validation, sensitivity, specificity, standardization, and scalability. The most common form of LB is cfDNA testing, but it can be influenced by improper specimen collection, anticoagulant use, and storage. To accurately interpret and make clinical decisions, it is crucial to understand the advantages and limitations of cfDNA analysis. LB shows promise as a tool for new precision medicine strategies in cancer treatment. In recent years, LB has been widely used in various studies, such as the development of a Multiple Genome Liquid Biopsy for Prostate Cancer in Whole Blood (PROSTest) (Modlin et al., 2024) and the rapid detection of ctDNA (circulating tumor DNA) in metastatic breast cancer using ultrasensitive and rapid circulating tumor DNA liquid biopsies with surface-restricted gene amplification on dispersible magnetic nanoelectrodes (Park et al., 2024). CtDNA has also emerged as a non-invasive tool to detect minimal residual disease and early detection of recurrence in early stage non-small cell lung cancer (NSCLC) (Tan et al., 2024). Free DNA has shown potential for preoperative malignancy risk assessment in ovarian masses, but further validation is needed through larger clinical studies (Gaillard et al., 2024). Li et al. (2024) developed a cfDNA-based multi-modal epigenetic sequencing analysis (MESA) method for the detection of CRC that captures and integrates cfDNA methylation and nucleosome occupancy. Overall, the detection of cfDNA is a growing trend in cancer research and provides new opportunities for non-invasive diagnosis and prognosis of cancer.

## 2 Common methods for methylation detection and research progress

Methylation detection methods have evolved significantly, offering a range of techniques to study DNA methylation patterns. Methylation assays are summarized in Figure 2. The advantages and disadvantages of methylation methods are summarized in Table 1.

**FIGURE 2:**
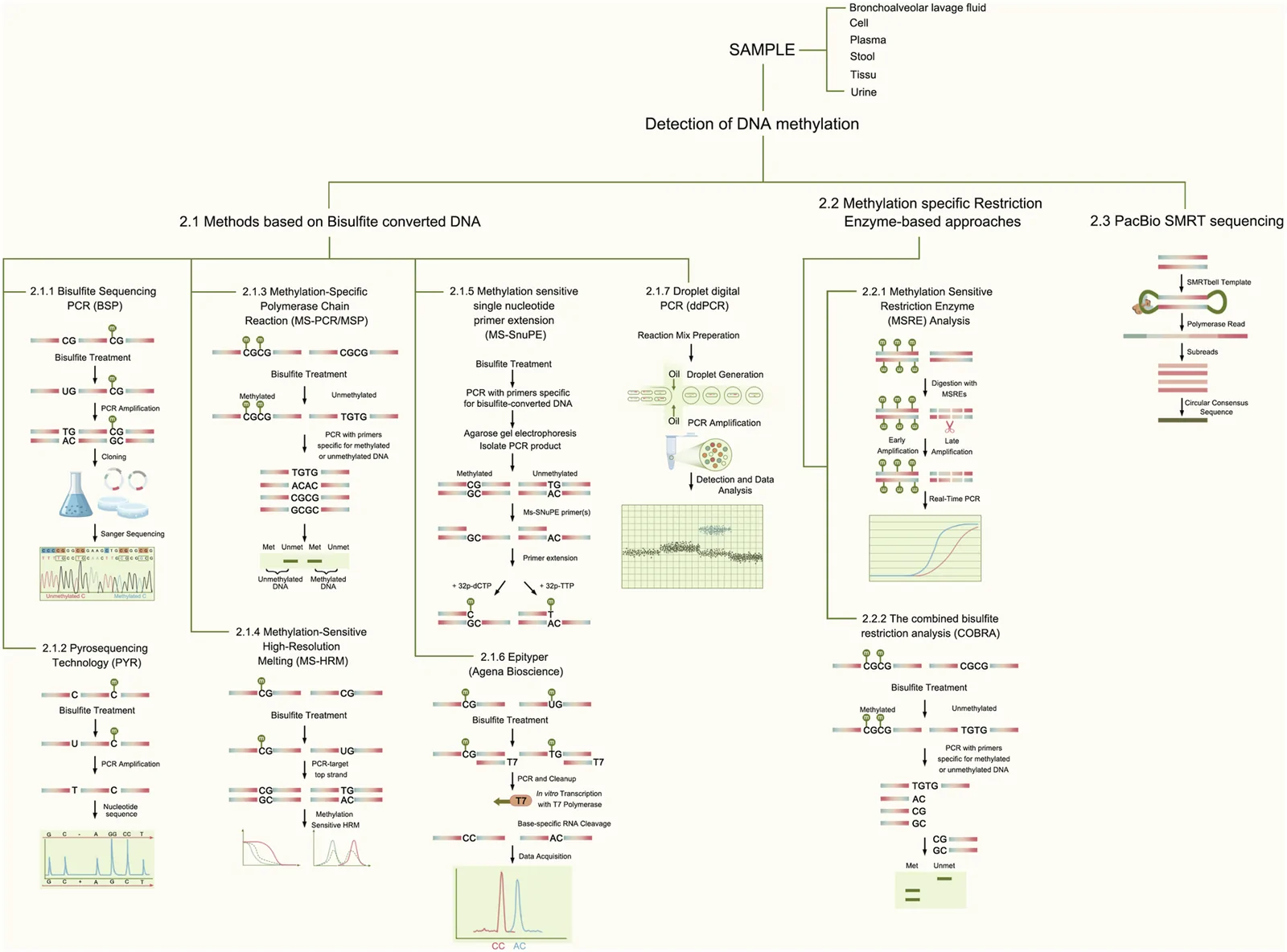
Methods Schematic for Methylation Detection. This figure describes the principles of common methods associated with gene methylation, including bisulfite sequencing PCR (BSP), pyrosequencing technology (PYR), methylation-specific polymerase chain reaction (MS-PCR/MSP), methylation-sensitive high-resolution melting (MS-HRM), methylation-sensitive single-nucleotide primer extension (MS-SnuPE), Epityper, Droplet digital PCR (ddPCR), methylation-sensitive restriction enzyme (MSRE) analysis, COBRA, and PacBio SMRT sequencing.

**TABLE 1:** Advantages and disadvantages of commonly used methylation methods.

Method	Advantages	Disadvantages
BSP	i. A simple method ii. It can be used for quick verification iii. The gold standard for DNA methylation analysis	i. Noisy sequencing results ii. Low sensitivity
PYR (Martisova et al., 2021)	i. It is easy to operate, repeatable, and inexpensive ii. Percent methylation can be calculated precisely as C/(C + T) iii. It provides excellent per-CpG resolution at any type of methylation site with highly normalized output iv. It has high sensitivity	i. Only shorter regions (approximately 50–60 base pairs) could be analyzed; otherwise, the sequencing data accuracy would be reduced ii. It is considered labor-intensive and relatively expensive iii. Low throughput
MS-PCR/MSP	i. Methylation analysis method for CGI only ii. High sensitivity	i. No information is provided regarding the methylation status of monocytosine ii. It cannot be used at sites with low numbers of CpGs iii. Primer design is difficult
MS-HRM	i. Methylated sites can be distinguished from unmethylated sites when only a small proportion of analyzed sample sites is methylated ii. Fast and cost-effective	i. Not only single CpGs can be analyzed
MS-SnuPE Martisova et al., (2021)	i. Single cytosine analysis without restriction enzyme or sequencing ii. Semiquantitative iii. Multiple CpGs can be analyzed per reaction when using multiple strategies	i. Two parallel reactions are required at each site and radiolabeled compounds are required
Epityper	i. High throughput, high reproducibility, a small number of samples required, high accuracy ii. High sensitivity	i. Expensive ii. Difficult to analyze sequences with peptide properties
Droplet digital PCR Hindson et al. (2011)	i. Rapid detection, in which a single DNA molecule can be amplified, screened, purified, and sequenced in a small volume of the sample on the same day ii. To validate biomarkers clinically applied to a low number of samples (cfDNA obtained from liquid biopsies) iii. High sensitivity	i. Designing primers is a laborious task (methylation-independent primers)
MSRE Analysis Šestáková et al. (2019)	i. MSRE analysis is a fast and simple method that does not require bisulfite conversion of DNA as other methods do ii. Less DNA is required, which makes primer design easier iii. High specificity	i. Not suitable for intermediate methylation regions ii. More expensive than PYR iii. Only specific restriction sites can be analyzed
COBRA	i. Sensitive quantitative methods, are most commonly used when detecting fully methylated and completely unmethylated samples ii. The method is relatively simple and does not require know ledge of the CpG site and sample sequence in advance iii. It requires a small sample size for the analysis of paraffin-embedded samples	i. Poor sensitivity compared to MSP. ii. Only specific restriction site methylation can be obtained, so negative detection cannot rule out the possibility of methylation in the sample DNA. iii. Sequence analysis is limited due to the use of enzymes and PCR.
PacBio SMRT sequencing	i. High accuracy ii. Fast sequencing	i. A small data volume, a chip is currently only up to 8 million wells ii. Single-molecule sequencing raw data has a high error rate and requires repeated sequencing to reduce the error rate iii. Sequencing is expensive and SMRT costs 6–7 times more than second-generation sequencing iv. Sequencers are costly and not suitable for small-scale tissue purchase

### 2.1 Methods based on bisulfite converted DNA

#### 2.1.1 Bisulfite sequencing PCR (BSP)

Genomic sequencing of bisulfite converted DNA is a popular technique for studying DNA methylation. Bisulfite treatment converts unmethylated cytosine to uracil, while leaving 5 mC unaffected. However, one major drawback of bisulfite treatment is DNA degradation, which limits its use for sequencing long DNA molecules. To overcome this, single-molecule sequencing methods like PacBio SMRT sequencing can be employed. We will discuss this method in more detail later. Commonly used tools for detecting DNA methylation in bisulfite-converted DNA include Bismark, BS-Seeker2, and BWA-Meth. Recently novel BSBolt methylation callers have emerged offering more accurate and faster methylation calls without the need for additional formatting (Farrell et al., 2021). High-throughput sequencing combined with bisulfite conversion is a widely used approach for analyzing genome-wide DNA methylation. In one method, biotinylated RNA probes are used to capture a small portion of the genome (approximately 4 Mb) before bisulfite conversion and sequencing (Morselli et al., 2021). This method efficiently handles a large amount of targeted bisulfite sequencing data, allowing for quantitative determination of known methylation statuses.

#### 2.1.2 Pyrosequencing technology (PYR)

The PYR method enables the accurate quantification of methylation frequencies at individual contiguous CpG sites, making it possible to detect subtle alterations in methylation levels. This is achieved by determining the proportion of C-T at a single site through the incorporation of C and T during sequence extension. Therefore, the PYR method can accurately detect methylation variants at different loci. It is a relatively simple and rapid method that provides comparable results. Additionally, PYR is suitable for analyzing CpG-poor or CpG-rich regions. The PYR assay consists of three steps (Harrington et al., 2013):① Biotinylated primers are used for PCR amplification and labeling.② Streptavidin separates the PCR products and hybridizes to sequencing primers.③ Sequencing is performed, with nucleotides added one by one according to the template sequence. This releases pyrophosphate (PPi) under the action of polymerase. Sulfatase is used to produce ATP together with adenosine phosphate (APS), and luciferase converts ATP to oxyfluorescein. The light intensity in the thermogram is presented as a peak. The PYR method can be used in combination with MSP to assess tumor tissue methylation in glioblastoma patients (Estival et al., 2019). It can also be employed to assess DNA methylation levels of microRNA-137 as a differential diagnosis between benign and malignant ascites (Bose et al., 2023). Furthermore, the PYR methods has been used to detect methylation of *CADM1* and *MAL* promoter regions in cervical cancer tissue samples as well as some basal methylation in healthy women (Mersakova et al., 2014).

#### 2.1.3 Methylation-specific polymerase chain reaction (MS-PCR/MSP)

MSP is a methylation detection method specifically designed for CGI. It utilizes two sets of primers for methylated and unmethylated alleles in selected regions. While gel electrophoresis is commonly used to assay PCR products, real-time PCR (qPCR) is now frequently employed for more sensitive results. The detect on of *TWIST1* and *NID2* gene methylation in urine sediment using MSP offers a highly sensitive and specific non-invasive method for detecting BCa (Renard et al., 2010). Currently, there are two methods for determining methylation products depending on the probes used for qPCR. The method using TaqMan probes is called MethyLight (Eads et al., 2000), and the method using SYBR Green I is called MethylQuant (Thomassin et al., 2004). MethyLight is a highly sensitive assay that can detect methylated alleles even in the presence of a 10,000-fold excess of unmethylated alleles. The development of this technology significantly enhances our ability to rapidly and accurately generate epigenetic profiles of tumor samples. Methylation levels of *SFRP1*, *SFRP2*, *SDC2*, and *PRIMA1* were determined using a MethyLight assay after cfDNA isolation and bisulfite conversion of samples (Barták et al., 2019). MethyLight can also quantitatively detect *SOX21* gene promoter methylation, serving as a fecal-based noninvasive biomarker for early diagnosis of colorectal cancer (Moradi et al., 2021). Furthermore, the MethyLight assay accurately detects *B3GAT2* and *ZNF793* methylation levels in endoscopic barrett’s esophagus (BE) samples (Yu et al., 2015). In validation experiments, the MS-HRM assay showed better sensitivity than the MethyLight assay (Wojdacz and Dobrovic, 2007). On the other hand, MethylQuant is an economically efficient and relatively simple technique that enables quantitative analysis of the methylation status of individual cytosines at specific locations in DNA (Dugast-Darzacq and Grange, 2009).

#### 2.1.4 Methylation-sensitive high-resolution melting (MS-HRM)

MS-HRM are utilized for comparing the melting curves of DNA sequences with varying base compositions. By analyzing these melting curves, we can identify differences between methylated and unmethylated DNA after bisulfite treatment. This method is highly efficient and can accurately distinguish between methylated and unmethylated sites, making it ideal for cases where only a small portion of the sample sites are methylated. To overcome PCR bias, specific primer design is crucial, especially after bisulfite treatment. Therefore, specific primer design requirements must be met for DNA sequences. Before conducting MS-HRM analysis, DNA must undergo bisulfite modification to differentiate the melting temperature between methylated and unmethylated templates. MS-HRM analysis relies on different melting temperatures (Tm) for C-G and A-T pairs. Tm refers to the temperature at which two DNA strands separate, resulting in a sudden decrease in fluorescence signal as the dye (e.g., SYBR Green) is released. The simplicity and high reproducibility of MS-HRM protocols have made it the preferred method for methylation assessment in various diagnostic and research applications (Wojdacz and Dobrovic, 2007). In a recent study (Liu et al., 2020), MS-HRM was employed to evaluate the methylation status of *CASK* (calmodulin-dependent serine protein kinase) in 296 tumor tissues. The combined use of MS-HRM and dMS-HRM allowed for the assessment of the extent and complexity of heterogeneous methylation revealing visible patterns.

#### 2.1.5 Methylation sensitive single nucleotide primer extension (MS-SnuPE)

MS-SnuPE is a commonly used method for analyzing cytosine methylation and quantification. It is performed after conversion and PCR amplification. In this method, the PCR product is used in a second reaction, that specifically targets the 3’ end before CpG after primer annealing. The resulting product is then separated by electrophoresis and analyzed using a phosphorimager (Gonzalgo and Jones, 1997). MS-SnuPE is particularly suitable for examining abnormal methylation patterns during tumorigenesis and monitoring changes induced by DNA methylation inhibitors (Gonzalgo and Liang, 2007). Previous studies have demonstrated the effectiveness of MS-SnuPE in investigating methylation markers such as *AGTR1, GALR1, SLC5A8, ZMYND10*, and *NTSR1* in non-small cell lung cancer (NSCLC) (Guo et al., 2015).

#### 2.1.6 Epityper (agena bioscience)

Epityper is an analytical method that utilizes base-specific cleavage and MALDI-TOF-MS (matrix-assisted laser desorption/ionization time-of-flight mass spectrometry). Although it requires expensive instruments, its high-throughput nature makes it ideal for testing a large number of samples. Epityper technology enables accurate, rapid, and reproducible quantification of DNA methylation status at single or multiple CpG positions in numerous samples. This technique can analyze DNA from various sample types, including liquid biopsies, fresh frozen tissues, and formalin-fixed paraffin-embedded tissues. Epityper has also been used to the detect *XXYLT1* methylation in lung adenocarcinoma (Zeng et al., 2021).

#### 2.1.7 Droplet digital PCR (ddPCR)

Droplet digital PCR (ddPCR) is a sequencing technique that uses water-oil emulsion droplet technology. PCR amplification occurs in each droplet, and the proportion of PCR-positive droplets in the original sample is then determined through analysis. Methylation targets are quantified using a standard mutation/SNP singleplex assay strategy in ddPCR analysis of bisulfite converted DNA samples. In the analysis of cell-free circulating DNA (cfDNA), the samples are bisulfite treated and the *SEPT9* and BMP3 methylation status is analyzed using a ddPCR system (Lima et al., 2023). Droplet digital methylation-specific PCR (ddMSP) is a combined method of ddPCR and MSP for the detection of methylation markers (*RASGRF1*, *CPXM1*, *HOXA10*, and *DACH1*) in breast cancer (Uehiro et al., 2016).

### 2.2 Methylation specific restriction enzyme-based approaches

#### 2.2.1 Methylation sensitive restriction enzyme (MSRE) analysis

Methylation-sensitive restriction enzyme (MSRE) is initially used to selectively digest unmethylated DNA while leaving methylated targets intact. The first method for assessing DNA methylation levels is MSRE analysis using methylation-sensitive restriction enzymes such as HpaII, AatII, and ClaI. These enzymes do not require bisulfite conversion of DNA (Cedar et al., 1979). Research has demonstrated that methylation-sensitive restriction enzyme bisulfite sequencing (MREBS) provides sufficient sequencing coverage and reduces measurable cytosine damage compared to whole-genome bisulfite sequencing (WGBS) and reduced representation bisulfite sequencing (RRBS) (Bonora et al., 2019). With the specificity of MSRE, the high amplification efficiency of Lambda-assisted RPA, and the self-amplification effect of CRISPR/Cas12a, as little as 0.05% of methylated DNA can be distinguished (Zhou et al., 2022). In a recent study, the researchers combined the high specificity of the methylation-sensitive restriction enzyme GlaI for methylase digestion with the high amplification efficiency of rolling circle amplification (RCA) to detect GlaI digestion products. The GlaI-RCA technology confirmed that methylated *Septin9* in colorectal cancer tissue samples correlated well with standard bisulfite sequencing (Dong et al., 2022).

#### 2.2.2 The combined bisulfite restriction analysis (COBRA)

COBRA is a technique that combines bisulfite conversion with restriction enzymes order to determine DNA methylation levels at specific gene loci in small amounts of genomic DNA. This technology offers ease of use, quantitative accuracy, and compatibility with paraffin sections (Xiong and Laird, 1997). In the COBRA assay, DNA is modified with sulfite esters and then amplified by PCR. The amplified products are then digested with endonucleases that can distinguish between methylated and unmethylated sequences. The COBRA assay allows for the analysis of methylation status at specific sites (Bilichak and Kovalchuk, 2017). Additionally, COBRA can be combined with bisulfite genome sequencing (BGS) to analyze two critical CpG sites in the *ROR2* promoter of endometrial cancer (EC) (Liu D. et al., 2021). Methylation levels at two positions in the promoter region of the *MGMT* gene were quantitatively analyzed using a combination of COBRA and native capillary gel electrophoresis (Goedecke et al., 2015).

### 2.3 PacBio SMRT sequencing

PacBio SMRT sequencing is a third-generation methylation assay. Currently, BSP is the mainstream method, but it has certain disadvantages, such as potential DNA degradation after bisulfite treatment. Additionally, BSP is unsuitable for sequencing long DNA molecules, especially with lower methylation levels that are more susceptible to degradation. Therefore, it is not well suited for thrid-generation sequencing. In contrast, PacBio SMRT sequencing can sequence a single DNA molecule and produce an average read length of 13.5 kb with 99.8% sequencing accuracy (Wenger et al., 2019). Choy et al., (2022) demonstrated the presence of long cfDNA in the plasma of hepatocellular carcinoma (HCC) using PacBio SMRT sequencing. They analyzed fragment size and methylation to determine the tissue origin of individual plasma DNA molecules. PacBio sequencing kinetics allows for direct detection of base modifications, such as N6-methyladenine (m6A) and N4-methylcytosine (m4C). Tse et al. (2021) developed a method for single-molecule real-time sequencing to examine 5 mC directly. This method achieves over 90% accuracy and sensitivity in detecting 5 mC at CpG sites in humans and mice based on polymerase signal characteristics. While PacBio SMRT sequencing technology is primarily used for methylation analysis of microbial genomes (Coy et al., 2020), further exploration is needed for sequencing analysis of methylation markers.

## 3 Early screening and detection of methylation markers in cancer

### 3.1 Digestive system cancer

#### 3.1.1 Colorectal cancer (CRC)

According to the 2020 Global Cancer Data Statistics, colorectal cancer ranks third among common cancers worldwide and second among cancer-related mortality worldwide (Sung et al., 2021). CRC is a heterogeneous disease, with patients exhibiting different phenotypes associated with varying prognoses and treatment regimens. Approximately 20% of CRC patients will develop metastases, resulting in a less than 5-year survival rate of only 14% with conventional treatment (Rumpold et al., 2020). The tumor stage at diagnosis is crucial for prognosis, highlighting the significance of considering CRC as an important condition. Carcinoembryonic antigen (CEA) is a well-known serum biomarker for CRC. However, it can also be elevated in various benign conditions, rendering it ineffective for early detection or screening. Many studies on methylation biomarkers have focused on plasma or fecal samples from CRC patients, such as blood samples from blood *KCNJ12* and *ZNF132* (Zhang et al., 2021), and stool samples from *BMP3, GATA4/GATA5, HLTF* and others (Liu et al., 2019). These studies have described DNA methylation biomarkers for CRC metastasis, which show promise for early clinical diagnosis. This article primarily outlines 11 methylation biomarkers (*ADHFE1, BMP3, CDX2, CYP24A1, DKK3, NSD2, SDC2, SEPT9, SFRP1, VIM*) that can be used for early screening of CRC. Additional biomarkers for CRC are listed in Table 2; Figure 3.

**TABLE 2:** Candidate biomarkers of methylation in early diagnosis of colorectal, hepatocellular, gastric, pancreatic and esophageal cancers.

Cancer type	Gene	Sample type	Type of marker	References
CRC	*ADHFE1*	Stool	Screening, Non-invasive diagnostic	Li et al. (2023)
	*ALX4*	Stool, Tissue	Screening	Sobhani et al. (2019), Sobanski et al. (2021)
	*BCL2*	Tissue	Screening	Zhu et al. (2011)
	*BMP3*	Plasma, Stool	Screening	Kisiel et al. (2019), Lima et al. (2023)
	*CDX2*	Tissue	Screening	Huang et al. (2020), Pan et al. (2022)
	*CYP24A1*	Tissue	Predictive	Wang et al. (2023)
	*DKK3*	Enterocyte	Predictive	Zhao et al. (2020)
	*HAND1*	Plasma	Diagnostic	Shavali et al. (2024)
	*KCNJ12*	Plasma	Diagnostic	Zhang et al. (2021a)
	*KRT20*	Tissue	Predictive	Lee et al. (2022)
	*NSD2*	Tissue	Predictive	Zhao et al. (2021)
	*PENK*	Plasma	Predictive	Sobhani et al. (2019)
	*PPP1R16B*	Plasma	Predictive	Bacolod et al. (2020)
	*PRIMA1*	Plasma	Predictive	Barták et al. (2019), Molnár (2020)
	*RARB*	Cell	Predictive	Fu et al. (2022)
	*SDC2*	Plasma, Stool, Urine	Screening, Diagnostic	Han et al. (2019), Bach et al. (2021), Zhang et al. (2021b), Xu et al. (2021), Ma et al. (2022b), Siri et al. (2022), Yuan et al. (2022), Yue et al. (2022)
	*SEPT9*	Plasma, Tissue, Urine, Stool	Screening, Diagnostic	Chen et al. (2019), Bach et al. (2021), Alizadeh-Sedigh et al. (2022), Lima et al. (2023)
	*SFRP1*	Plasma, Stool	Screening	(Liu et al., 2019; Pasha et al., 2019; Barták et al., 2019)
	*SFRP2*	Plasma, Stool, Tissue	Screening, Diagnostic	Barták et al. (2019), Jamialahmadi et al. (2021), Lin et al. (2022)
	*SOCS3*	Cell	Predictive	Liu et al. (2023)
	*SOX21*	Plasma, Stool	Screening, Diagnostic	Moradi et al. (2021)
	*VIM*	Plasma, Stool	Screening	Yuan et al. (2022)
	*WIF1*	Stool	Screening	Ma et al. (2022b)
	*ZNF132*	Plasma	Screening	Zhang et al. (2021a)
HCC	*DBX2*	Cell	Diagnostic	Wu et al. (2020a)
	*GSTP1*	Tissue	Screening, Diagnostic	Schnekenburger et al. (2014)
	*mSEPT9*	Plasma	Screening, Diagnostic	Oussalah et al. (2018), Li et al. (2020a)
GC	*CDH1*	Tissue, Plasma	Predictive	Zeng et al. (2015)
	*DAPK*	Tissue, Plasma	Predictive	Jia et al. (2016)
	*LINE-1*	Tissue	Predictive	Baba et al. (2014)
	*MGMT*	Tissue	Predictive	Yuan et al. (2017)
	*P16*	Tissue, Cell, Plasma	Predictive	Spagnol et al. (2022)
	*PCDH10*	Plasma	Screening	Schneider et al. (2015)
	*RASSF1A*	Tissue, Plasma	Screening	Nemtsova et al. (2017)
	*RASSF2*	Tissue, Stool	Predictive	Zhou et al. (2019a)
	*RPRM*	Plasma	Predictive	Amigo et al. (2018)
	*RUNX3*	Tissue, Plasma	Predictive	Liu et al. (2016)
PC	*ADAMTS1*	Plasma, Pancreatic juice	Screening, Diagnostic	Eissa et al. (2019)
	*BNC1*	Plasma, Pancreatic juice	Screening, Diagnostic	Eissa et al. (2019)
BE	*B3GAT2*	Tissue	Screening	Yu et al. (2015a)
	*CCNA1*	Tissue	Screening	Moinova et al. (2018)
	*TFPI2*	Tissue	Screening, Diagnostic	Chettouh et al. (2018)
	*TWIST1*	Tissue	Screening	Chettouh et al. (2018)
	*VIM*	Tissue	Screening	Moinova et al. (2018)
	*ZNF793*	Tissue	Screening	Yu et al. (2015a)

**FIGURE 3:**
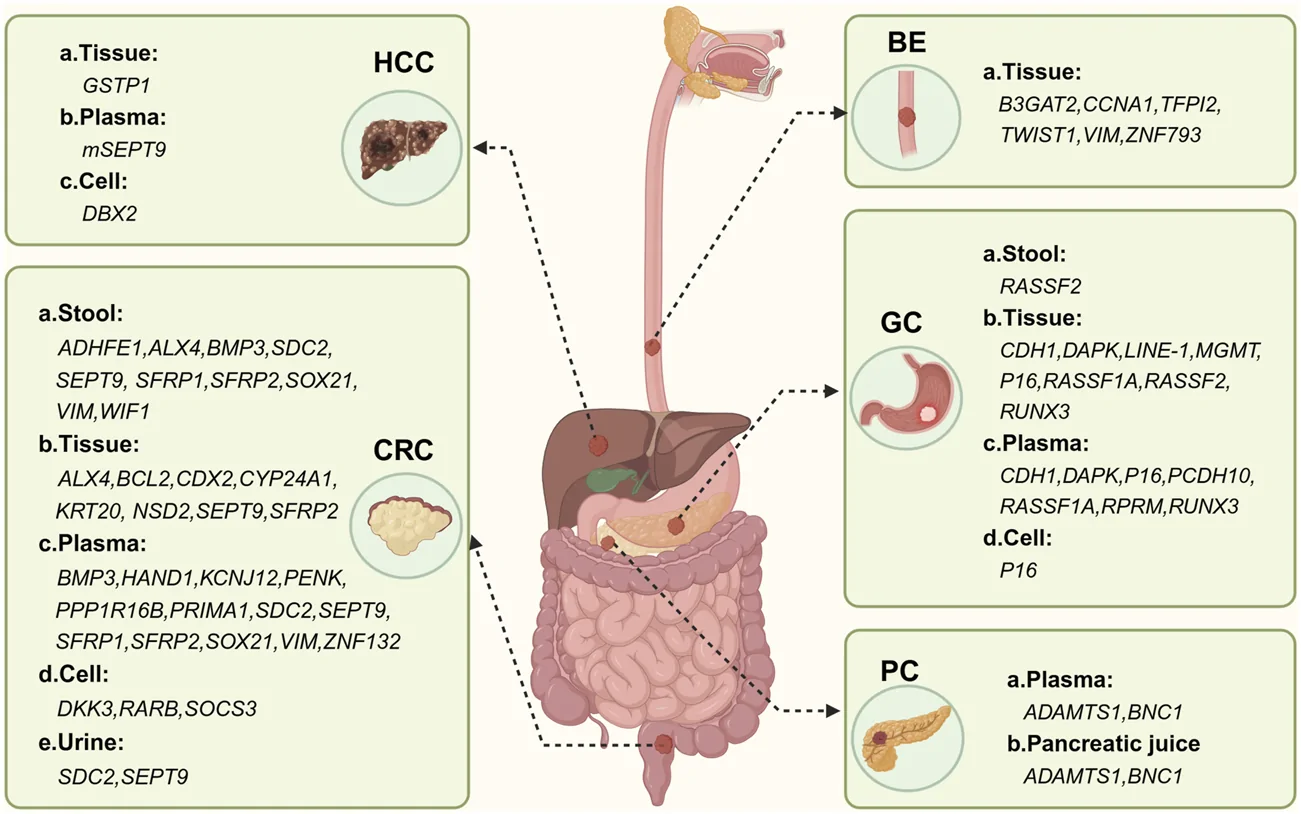
Methylation-based biomarker candidates in different samples for digestive system cancer diagnosis. Abbreviations: HCC: a. Tissue:*GSTP1* (glutathione S-transferase pi 1); b. Plasma: *mSEPT9*; c. cell: *DBX2* (developing brain homeobox 2). CRC: a. Stool: *ADHFE1* (alcoholdehydrogenase, ironcontaining, 1), *ALX4* (ALX homeobox 4), *BMP3* (bone morphogenetic protein 3), *SDC2* (syndecan 2), *SEPT9* (septin 9), *SFRP1* (secreted frizzled-related protein 1), *SFRP2* (secreted frizzled-related protein 2), *SOX21* (sex determining region Y box 21), *VIM* (vimentin), *WIF1* (WNT inhibitory factor 1); b. Tissue: *ALX4* (ALX homeobox 4), *BCL2* (B-cell CLL/lymphoma 2), *CDX2* (caudal type homeobox 2), *CYP24A1* (cytochrome P450, family 24, subfamily A, polypeptide 1), *KRT20*, *NSD2* (Wolf-Hirschhorn syndrome candidate 1), *SEPT9*, *SFRP2;* c. Plasma: *BMP3, HAND1* (heart and neural crest derivatives expressed 1), *KCNJ12* (potassium channel, inwardly rectifying subfamily J, member 12), *PENK* (proenkephalin), *PPP1R16B* (protein phosphatase 1, regulatory subunit 16B), *PRIMA1* (proline rich membrane anchor 1), *SDC2* (syndecan 2), *SEPT9*, *SFRP1*, *SFRP2*, *SOX21*, *VIM;* d. Cell: *DKK3* (dickkopf WNT signaling pathway inhibitor 3), *RARB* (retinoic acid receptor, beta), *SOCS3* (suppressor of cytokine signaling 3); e. Urine: *SDC2*, *SEPT9*. BE: a. Tissue: *B3GAT2* (beta-1,3-glucuronyltransferase 2), *CCNA1* (cyclin A1), *TFPI2* (tissue factor pathway inhibitor 2), *TWIST1* (twist family bHLH transcription factor 1), *VIM*, *ZNF793* (zinc finger protein 793). GC: a. Stool: *RASSF2* (Ras association domain family member 2)*;*b. Tissue: *CDH1* (cadherin 1), *DAPK* (death-associated protein kinase 1), *LINE-1*, *MGMT* (O-6-methylguanine-DNA methyltransferase), *P16* (cyclin-dependent kinase inhibitor 2A), *RASSF1A* (Ras association domain family member 1), *RASSF2*, *RUNX3* (runt-related transcription factor 3); c. Plasma: *CDH1*, *DAPK* (death-associated protein kinase 1), *P16*, PCDH10 (protocadherin 10), *RASSF1A*, RPRM(reprimo), *RUNX3* (runt-related transcription factor 3); d. Cell: *P16.* PDA: a. Plasma: *ADAMTS1* (ADAM metallopeptidase with thrombospondin type 1 motif, 1), *BNC1* (basonuclin 1); b. Pancreatic juice: *ADAMTS1*, *BNC1*. Figure created with BioRender.com.

Hypermethylation of *ADHFE1* (Alcohol dehydrogenase iron containing 1) promotes the proliferation of CRC cells by regulating cell cycle progression (Hu et al., 2019). This hypermethylation can serve as an early screening indicator, and restoring *ADHFE1* may offer a new treatment option for CRC patients. A recent study has demonstrated that the combined detection of *ADHFE1/SDC2/PPP2R5C* methylation could potentially be used as a non-invasive diagnostic approach for CRC and screening for precancerous lesions (Li et al., 2023). *BMP3* (bone morphogenetic protein 3) belongs to the transforming growth factor-β (*TGFB*) superfamily. It is used for multi-target fecal DNA detection (Tepus and Yau, 2020). The promoters of *BMP3* and *SEPT9* (septin 9) in plasma samples can serve as indicators for early detection and screening of CRC in the population (Lima et al., 2023). Methylation levels of *BMP3* and *VAV3* in stool samples are highly specific for CRC surveillance (Kisiel et al., 2019). BRAF transcription factor *CDX2* (caudal type homeobox 2) undergoes DNA methylation silencing in proximal colon cancer (Yang et al., 2024). *JARID1B* regulates *CDX2* expression by demethylation of *H3K4me3*, which inhibits upstream methylation and promotes cell proliferation in CRC through the Wnt/beta-catenin pathway (Huang et al., 2020). *CDX2* is also inhibited by *SMYD2*-mediated *MEX3A* to enhance CRC progression (Pan et al., 2022), Decreased expression of *CDX2* is more dependent on the CpG island hypermethylation phenotype (CIMP-H) (Lee et al., 2022). The methylation status of *CYP2R1* (cytochrome P450) can influence contributors to vitamin D serum levels and predict response to vitamin D supplementation. It has been shown that elevated serum levels of 25-hydroxyvitamin D (25 (OH) D) result in impaired methylation of *CYP24A1* (Forouhari et al., 2023). Recent studies have shown that cumulative methylation levels at CpG sites in *CYP24A1* are inversely associated with CRC risk (Wang et al., 2023). In most human cancer cells, the expression of *DKK3* (Dickkopf-related protein 3) is epigenetically silenced by methylation of CpG islands in promoters (Nozaki et al., 2001). The mRNA expression of *DKK3* is inversely correlated with its promoter methylation and may inhibit tumorigenesis and serve as a molecular diagnostic target (Zhao et al., 2020). *NSD2* (Histone methyl transferase nuclear receptor-binding SET domain 2) is a crucial enzyme that catalyzes histone H3 lysine 36 dimethylation (*H3K36me2*). Elevated expression of *NSD2* mRNA has been observed in colon cancer. Furthermore, silencing or knocking down *NSD2* leads to a significant reduction in the activation of multiple oncogenes (*ADAM2*, *EGFR*, *Sox2*, *Bcl-2*, *SYK*, and *MET*). This suggests that *NSD2* may serve as a novel target for detection and therapeutic intervention (Zhao et al., 2021). *SDC2* (syndecan 2) is a transmembrane heparan sulfate proteoglycan. It encodes the *SDC2* protein, which plays a role in the interaction regulation of cell-environment interactions. *SDC2* affects cell proliferation, migration, invasion, and tumor development (Hua et al., 2020). The methylation status of the *SDC2* promoter in blood samples can be detected using the methylation quantitative endonuclease resistance DNA assay (MethyQESD). This assay has shown high accuracy in differentiating CRC patients at an early stage of the disease (Siri et al., 2022). *SDC2* can be combined with many biomarkers for methylation detection. For example, *SDC2-SEPTIN9-BCAT1* combined with ctDNA methylation demonstrates high sensitivity and specificity (Xu et al., 2021). Similarly, the combined detection of *SDC2-SEPTIN9-VIM* has the potential to serve as a biomarker for early diagnosis of CRC (Yuan et al., 2022). The detection of aberrantly methylated DNA in feces has proven to be an effective early screening method for CRC. The combined detection of methylated *SDC2-TFPI2* in feces holds promise as one of the effective and noninvasive tools for early CRC screening (Zhang et al., 2021). Additionally, the combined detection of *SDC2-ADHFE1-PPP2R5C* methylation could serve as a potentially non-invasive diagnostic method for CRC and precancerous lesion screening (Li et al., 2023). In clinical applications, the combined *SDC2-NDRG4* methylation detection assay has shown excellent performance in the detection of CRC and advanced adenomas (Zhang et al., 2024). A study found that the sensitivity of CRC detection increased to 87.25% when methylated *SDC2-A* and methylated *SDC2-B* were combined. When using methylated *SDC2-A* alone, the sensitivity was 85.29%, and when using methylated *SDC2-B* alone, the sensitivity was 83.33% (Ma et al., 2022a). *SDC2-SFRP2* methylation was detected using quantitative PCR (qPCR) or methylation-specific quantitative PCR (MSP) (Lin et al., 2022). There are also new methods for detecting *SDC2*, such as linear target enrichment quantitative methylation-specific real-time PCR (LTE-qMSP), which is highly specific for early detection of *SDC2* methylation in fecal DNA in CRC patients (Han et al., 2019). Droplet digital PCR (ddPCR) indicated that the sensitivity and specificity of *SDC2* gene methylation detection could be reduced to 0.1% methylation level and 5 ng methylated DNA input (Yue et al., 2022). The ColoCaller test allows for simultaneous detection of the methylation status of *SDC2, TFPI2, WIF1,* and *NDRG4* genes in fecal DNA, optimizing and screening for colorectal cancer (Ma et al., 2022b). Marker groups consisting of *SDC2* and *SEPT9* were detected in urine sediment of CRC cases with a specificity of 70%, showing promise for non-invasive CRC detection through urine liquid biopsy (Bach et al., 2021). *SEPT9* plays a crucial role in cell division, and its hypermethylation process is linked to carcinogenesis and can alter the conformation of gene sequences. Methylated CpG sites bind to methylated CpG-binding proteins and interact with other proteins to form abnormal chromatin, which inhibits gene expression (Wasserkort et al., 2013). Additionally, the combination of plasma biomarkers *SEPT9-BMP3* has been used for early screening of CRC in a Brazilian population (Lima et al., 2023). The methylation panel consisting of *SEPT9-SPG20-FBN1* has higher diagnostic value compared to individual biomarkers or other combinations. This panel serves as a simple noninvasive methylation marker for early detection of CRC (Alizadeh-Sedigh et al., 2022). Studies have shown that 90% of CRC patients (27 out of 30) exhibit hypermethylation in the *SEPT9* gene, while 83% (25 out of 30) show hypermethylation in the *HAND1* gene. This emphasizes the potential of *SEPT9* and *HAND1* methylation as promising biomarkers for the diagnosis of CRC, leading to early detection and improved patient outcomes (Shavali et al., 2024). Moreover, *SEPT9* in urine (Bach et al., 2021) and stool (Chen et al., 2019) samples can also serve as methylation markers for early CRC screening. In recent years, a noninvasive test called methylated CpG tandem amplification and sequencing (MCTA-Seq) has been developed for CRC, specifically for detecting *SEPT9*. *SFRP1* (secreted frizzled-related protein 1) promoter DNA hypermethylation reduces the mRNA expression levels of *SFRP1*, and it is regulated by *H3K27me3* and DNA methylation (Wu et al., 2023). The combined detection of the *SFRP1-RUNX3-CEA* panel may serve as noninvasive biomarkers for early detection and diagnosis of CRC (Pasha et al., 2019). MethyLight assay can be used to determine the methylation status of *SFRP1* (Barták et al., 2019), *SFRP2* (Li et al., 2019), and *SOX21* (Moradi et al., 2021). *VIM* (vimentin) and *SEPT9* are epigenetic markers in CRC (Bacolod et al., 2020). The use of methylation markers for three genes, *VIM-SEPTIN9-SDC2*, enhances signal detection and can improve the accuracy of early CRC diagnosis. These markers show great potential for clinical application (Yuan et al., 2022).

#### 3.1.2 Hepatocellular carcinoma (HCC)

Global cancer statistics in 2020 revealed that liver cancer ranks as the sixth most prevalent cancer, with 905,677 new cases per year (Sung et al., 2021). HCC is a growing health concern and the second leading cause of cancer-related deaths worldwide, and it is the ninth most common cancer in women and the fifth most common cancer in men (Cao et al., 2021). Risk factors for HCC include cirrhosis, hepatitis B virus (HBV) infection, hepatitis C virus (HCV) infection, metabolic syndrome, hemochromatosis, α1-antitrypsin deficiency, and alcohol consumption. The development of HCC typically follows the path of hepatitis → cirrhosis → precancerous lesions → hepatocellular carcinoma, with 80%–90% of patients progressing through this sequence. Patients with early stage (I) liver cancer infected with hepatitis B virus have a significantly higher 5-year survival rate compared to those with advanced stage (III) liver cancer. However, HCC is often asymptomatic in the early stage, and it is frequently detected in the late stage. In recent years, alterations in DNA methylation have been proposed as “liquid biopsies”, which ctDNA, circulating tumor cells (CTC), circulating miRNAs, and exosomes (Li et al., 2018). Among these, ctDNA shows potential as an early diagnostic tool for HCC and can be found in a various early stage tumers such as colorectal cancer, ovarian cancer, lung cancer and breast cancer (Phallen et al., 2017). Currently, about 50% of HCC biomarkers are being investigated at the gene methylation level, with ctDNA methylation profiles in plasma being one of the most extensively studied areas in HCC liquid biopsies. Epigenetically altered genes are often associated with specific etiologic factors. For example, alpha-fetoprotein (AFP), miR-122, and circulating *TERT* promoter mutations are biomarkers used for diagnostic screening of HBV-related HCC (Trung et al., 2020). The following provides an overview of two well-studied methylation biomarkers that can be used for early HCC screening in recent years. Additional biomarkers are presented in Table 2; Figure 3.

*GSTP1* (glutathione S-transferase pi 1) is a key metabolic enzyme gene involved in phase II metabolism of various carcinogens. It plays an important role in maintaining cellular metabolism by decomposing carcinogens, protecting against oxidative damage, and the development of cancer cells. *GSTP1* has a large CpG-rich region that is frequently affected by methylation. It is frequently silenced by CpG island promoter hypermethylation in liver cancer, prostate cancer, breast cancer, and others. Some studies have shown that *GSTP1* methylation can serve as a diagnostic tool for HCC with a sensitivity of approximately 50%–75% and a specificity of 70%–91% (Gurioli et al., 2018). When combined with other biomarkers such as *APC* or *SOCS* (Liu et al., 2015), it can be used for early cancer detection in individuals who are genetically predisposed or exposed to carcinogens (Schnekenburger et al., 2014). Plasma *mSEPT9* is a novel circulating free DNA epigenetic biomarker for the diagnosis of HCC (Oussalah et al., 2018). In a study by Li et al. (2020), the diagnostic performance of plasma *SEPT9* for HCC was assessed using methylation-specific fluorescent quantitative PCR. The sensitivity of *SEPT9* for detecting early HCC increased to 91.3% and 87.7% when combined with serum AFP. The combination of *mSEPT9-AFP-PIVKA-II* is optimal compared to any single marker and may be useful for opportunistic screening of HCC in the cirrhotic population (Zheng et al., 2023).

#### 3.1.3 Gastric cancer (GC)

According to World Health Organization (WHO) statistics in 2020, gastric cancer is the fifth most common cancer worldwide and the fourth leading cause of cancer death (Sung et al., 2021). The development of GC involves multiple factors, including *Helicobacter pylori* and Epstein-Barr virus infections, exposure to carcinogens, suppression of tumor suppression genes, abnormal cell apoptosis, and disruption of cell cycle regulation. The progression of GC can be observed through various stages, starting from superficial gastritis, then progressing to atrophic gastritis, intestinal metaplasia, gastric epithelial dysplasia, and finally gastric cancer. Early detection of GC, either in its pre-malignant or early stages, can greatly improve the 5-year survival rate. Some countries with high GC incidence, such as Japan and Korea (Xia and Aadam, 2022), have established screening programs to enhance early detection and overall survival rates. DNA methylation, a process that involves specific genetic and epigenetic changes in the early stages of GC, can be detected in various bodily fluids, such as blood, gastric juice, and feces. Therefore, DNA methylation biomarkers can serve as indicators for the pathogenesis and early screening of GC. This article will outline five potential methylation biomarkers for the early screening of GC, with additional details presented in Table 2; Figure 3.

DNMT3A interacts with YAP/TAZ and recognizes CpG islands within the *CDH1* promoter. This interaction leads to hypermethylation of the *CDH1* (cadherin 1) promoter, resulting in the transcriptional silencing of *CDH1* and the acceleration of epithelial mesenchymal transition (Xu et al., 2024). *CDH1* hypermethylation levels are higher in the gastric mucosa adjacent to cancer compared to normal gastric mucosa, and this hypermethylation is closely associated with *H. pylori* (HP) status (Zeng et al., 2015) Pyrosequencing is commonly used to quantify the methylation of the *CDH1* gene. In gastric mucosa, *LINE-1* hypomethylation is linked to *H. pylori* infection, and *LINE-1* methylation levels hold promise as a surrogate marker for epigenetic defects in gastric carcinogenesis (Kosumi et al., 2015). *MGMT* is a DNA repair enzyme that plays a role in maintaining gene stability directly reversing guanine O6 alkylation through methyl groups, which helps prevent tumorigenesis. However, *MGMT* gene methylation is involved in GC development. A meta-analysis of *MGMT* promoter methylation and GC suggests that *MGMT* may be an important biomarker for GC progression (Yuan et al., 2017). The *P16* gene (cyclin-dependent kinase inhibitor 2A) located in the chromosome 9P21 region and serve as a key regulator in the cell cycle. The positive rate of *P16* gene methylation is significantly higher in lymph node metastasis of GC Considered the second largest tumor suppressor gene after *TP53*, the *P16* gene belongs to the *INK4* gene family. Methylation of the *P16* gene promoter has relatively low sensitivity but high specificity for the diagnosis of GC (Peng et al., 2014). The Reprimo gene family consists of single exonic genes found only in the vertebrate lineage, including *RPRM* and *RPRM-like* (*RPRML*). Loss of *RPRM* expression is associated with increased proliferation and growth of GC cells, and this poorly understood gene family has the potential for new translational applications as biomarkers for early screening in the future (Amigo et al., 2018).

#### 3.1.4 Pancreatic cancer (PC)

Pancreatic cancer is a highly aggressive malignancy that is often not detected until it has reached an advanced stage. According to changes in cancer survival rates in China from 2003 to 2015, the 5-year standardized survival rate for PC is only 7.2% (Zeng et al., 2018). Unfortunately, there are currently no widely used early diagnostic tests for PC, and only a few prognostic tests are available. This is largely due to the lack of reliable biomarkers that can accurately detect the early progression of the disease. However, there is promising research being conducted on the use of circulating tumor DNA (ctDNA) for the early diagnosis of PC. At present, many studies are currently analyzing the methylation of various genes and exploring the possibility of combining multiple candidate genes to create combined biomarkers. These biomarkers can be detected in body fluids such as pancreatic juice and blood. One such promising biomarker is *BNC1* (basonuclin 1), which has shown potential for the early diagnosis of PC. Combining the cfDNA methylation of *BNC1* with *ADAMTS1* (ADAM metallopeptidase with thrombospondin type 1 motif 1) has proven to be a powerful tool for the early detection of PC (Eissa et al., 2019).

#### 3.1.5 Esophageal cancer

DNA methylation can be used as an early detection marker for esophageal cancer. For example, methylation of *HIN1, TFPI2, DACH1,* and *SOX17* (Ma et al., 2016) has shown promising results. Barrett’s esophagus (BE) is a precancerous lesion that occurs due to gastroesophageal reflux. In BE, the normal esophageal squamous epithelium is replaced by intestinal metaplasia, which is believed to be a precursor of esophageal adenocarcinoma (EAC). DNA methylation is a common epigenetic change in BE. Methylation of *B3GAT2* (beta-1,3-glucuronyltransferase 2), *CCNA1*, *TFPI2*, *TWIST1*, *VIM*, and *ZNF793* (Zinc finger protein 793) can be confirmed using MethyLight PCR and Cytosponge, a non-endoscopic tool (Chettouh et al., 2018). Among these, *TFPI2* has shown promise as a diagnostic biomarker for BE. Combined detection of *B3GAT2* and *ZNF793* may serve as an early biomarker for noninvasive detection of BE (Yu M. et al., 2015). Additionally, a combination of *CCNA1* (cyclin A1) and *VIM* biomarkers has demonstrated a sensitivity of 90.3% and a specificity of 91.7% in detecting BE metaplasia following DNA methylation (Moinova et al., 2018). Methylation of *TFPI2* (Tissue factor pathway inhibitor 2) and *TWIST1* (Twist family bHLH transcription factor 1) can also be detected early using Cytosponge non-endoscopic cell collection devices (Chettouh et al., 2018).

### 3.2 Respiratory cancer

Lung cancer (LC) is the most common cancer and the leading cause of cancer death in men. However, there are currently limited effective and practical methods available for its early detection. According to the latest World Health Organization statistics in 2020, lung cancer is the second leading cause of cancer-related death worldwide and ranks first in cancer-related mortality worldwide (Sung et al., 2021). The early symptoms of LC are not typical, and by the time symptoms appear, the cancer has often already progressed significantly. Over the last 2 decades, low-dose computed tomography (LDCT) has been the most commonly used method for LC screening (Liang et al., 2019). However, the high sensitivity of LDCT can result in frequent false positives, and further investigation is needed to address its limitations. Therefore, there is a pressing need to explore biomarkers that can improve the early detection rate of LC. Abnormal DNA methylation typically occurs in the early stages of tumorigenesis and can provide new insights for the early detection of LC. There are two main types of LC: non-small cell lung cancer (NSCLC) and small cell lung cancer (SCLC). NSCLC is more closely associated with methylation. Recent studies have shown that liquid biopsy can be performed to detect lung cancer-related biomarkers in various bodily fluids, including plasma, sputum, bronchial fluid (such as bronchoalveolar lavage fluid, bronchial aspirate, and bronchial washings), and urine (Li et al., 2022). In patients with malignant tumors, ctDNA mainly consists of DNA from primary or metastatic tumor cells. The DNA is released into the blood from both healthy cells and tumor cells due to necrosis or apoptosis. CtDNA methylation in the blood may serve as an effective biomarker for LC. Sputum cytology is now known to have very low sensitivity for detecting LC in the early stages (Su et al., 2016). However, DNA hypermethylation status in sputum has been detected in LC patients in recent years (Hubers et al., 2017). Similarly, bronchoalveolar lavage fluid (BALF), bronchial aspirates, and bronchial washings, which are obtained by fiberoptic bronchoscopy, have characteristics similar to those of the LC tumor microenvironment and are less invasive. For example, a combination of *SHOX2* and *RASSF1A* in bronchoalveolar lavage fluid has been shown to perform methylation analysis, with higher sensitivity and specificity compared to conventional cytology and serum CEA in the diagnosis of LC (Zhang et al., 2017). Additionally, DNA methylation of *SHOX2* in bronchial aspirates can distinguish LC patients from healthy individuals with high sensitivity and specificity (Schmidt et al., 2010). DNA methylation biomarkers in bronchial washings can be used for early diagnosis of NSCLC (Um et al., 2018). Recent studies have also found that DNA methylated promoters of *CDO1*, *TAC1*, *HOXA9* and *SOX17* can be detected in the urine of patients with NSCLC (Liu et al., 2020; Bach et al., 2022). The following mainly outlines six methylation biomarkers that may be used as early screening biomarkers for NSCLC, with additional information provided in Table 3; Figure 4.

**TABLE 3:** Candidate biomarkers of methylation in early diagnosis of lung cancer.

Cancer type	Gene	Sample type	Type of marker	References
LC	*APC*	Plasma, Sputum, Bronchoalveolar lavage fluid	Screening, Diagnostic	Wang et al. (2017), Liu et al. (2021b), Borg et al. (2023)
	*CDH1*	Plasma, Bronchoalveolar lavage fluid	Screening	Topaloglu et al. (2004), Krishnamurthy et al. (2019)
	*CDH13*	Plasma	Screening, Diagnostic	Wang et al. (2017)
	*CDO1*	Plasma, Urine, Sputum	Screening, Diagnostic	Liu et al. (2017), Ooki et al. (2017), Liu et al. (2020b)
	*HOXA9*	Plasma, Urine, Sputum	Diagnostic	Hwang et al. (2011), Abou-Zeid et al. (2023)
	*MGMT*	Plasma, Sputum, Bronchoalveolar lavage fluid	Screening	Liu et al. (2017), Wang et al. (2017), Chen et al. (2021)
	*P16*	Plasma, Sputum, Bronchoalveolar lavage fluid	Screening, Diagnostic	Liu et al. (2017), Tuo et al. (2018)
	*RASSF1A*	Plasma, Sputum	Screening	Liu et al. (2017), Palanca-Ballester et al. (2021)
	*SHOX2*	Plasma, Bronchoalveolar lavage fluid, Bronchial aspirate	Predictive, Diagnostic	Weiss et al. (2017), Liu et al. (2021c), Palanca-Ballester et al. (2021)
	*SOX17*	Sputum	Screening	Liu et al. (2017)
	*TAC1*	Sputum	Screening	Liu et al. (2017)

**FIGURE 4:**
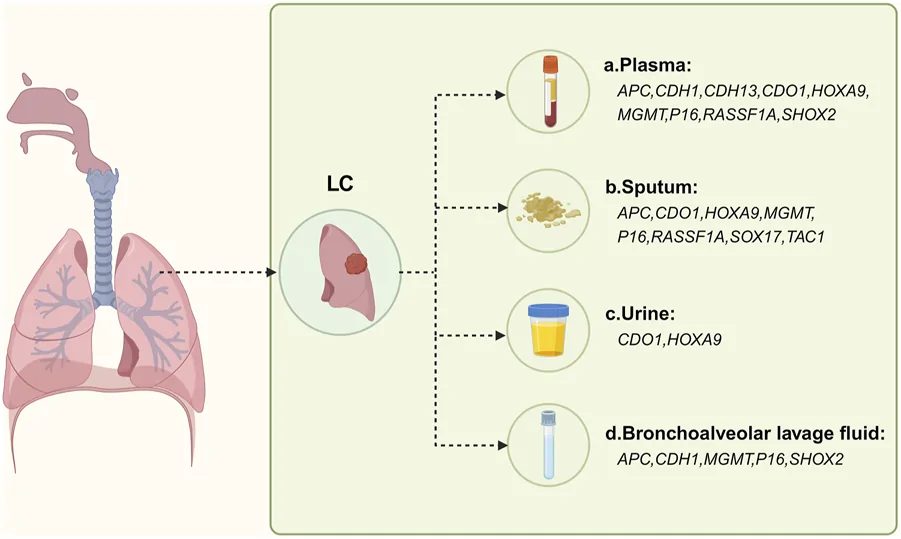
Methylation-based biomarker candidates in different samples for respiratory cancer diagnosis. Abbreviations: LC: a. Plasma: *APC* (adenomatous polyposis coli), *CDH1* (cadherin 1), *CDH13* (cadherin 13), *CDO1* (cysteine dioxygenase type 1), *HOXA9* (homeobox A9), *MGMT* (O-6-methylguanine-DNA methyltransferase), *P16* (cyclin-dependent kinase inhibitor 2A), *RASSF1A* (Ras association domain family member 1), *SHOX2* (short stature homeobox 2); b. Sputum: *APC*, *CDO1*, *HOXA9*, *MGMT*, *P16*, *RASSF1A*, *SOX17* (sex determining region Y-box 17), *TAC1* (tachykinin precursor 1); c. Urine: *CDO1*, *HOXA9*; d. Bronchoalveolar lavage fluid: *APC*, *CDH1*, *MGMT*, *P16*, *SHOX2.* Figure created with BioRender.com.

*APC* (adenomatous polyposis coli) encodes a tumor suppressor protein that acts as an antagonist of the Wnt signaling pathway. Defects in this gene result in familial adenomatous polyposis (FAP), an autosomal dominant precancerous condition that often progresses to malignancy. The methylation of the *APC* gene promoter is a potential biomarker for the diagnosis of LC. It is particularly useful when detected in serum, sputum, or bronchial lavage fluid of patients with NSCLC, as it exhibits high specificity. However, its low sensitivity makes it unsuitable for lung cancer screening in the general population (Liu et al., 2021). *CDH1* (cadherin 1) is a calcium-dependent cell-cell adhesion glycoprotein that is believed to promote cancer progression through proliferation, invasion, and/or metastasis. A meta-analysis (Yu et al., 2015) revealed higher methylation rates of *CDH1* in stages III and IV compared to stages I and II, suggesting its potential as an early screening biomarker for LC. The combination of three genes, *CDO1* (cysteine dioxygenase type 1), *HOXA9* (homeobox A9), and *TAC1* (tachykinin, precursor 1) has shown great promise in both the early diagnosis and staging of NSCLC (Wrangle et al., 2014). Hypermethylation of CpG island-rich gene promoters is one of the epigenetic mechanisms involved in carcinogenesis. It is considered a potential biomarker for LC. *HOXA9* is one of the genes most associated with LC through this mechanism. Hypermethylation of the *HOXA9* promoter leads to its transcriptional inactivation in LC (Rhee et al., 2015). The low expression of *HOXA9* is associated with epithelial-mesenchymal transition (EMT), which may be a feature of tumor invasion in NSCLC patients. Abou-Zeid et al., (2023) studied a potential diagnostic for NSCLC that combines *HOXA9* gene promoter methylation with *SOX2* and *HV2* genes in cfDNA. They used PYR and real-time PCR to detect the methylation of the three promoters in blood samples, aiming to determine whether they could serve as non-invasive diagnostic markers for NSCLC. Paschidis et al. (2022) observed *MGMT* promoter methylation in 30% of patients using methylation-sensitive high-resolution melting analysis (MS-HRM) and bisulfite pyrosequencing or Sanger sequencing analysis. Methylation of the *MGMT* gene promoter can be used as a biomarker for the early diagnosis of LC. A recent meta-analysis (Chen et al., 2021) showed that *MGMT* is hypermethylated in the plasma and bronchoalveolar lavage fluid of LC patients. *RASSF1A* (Ras association domain-containing protein 1A) is a well-studied tumor suppressor gene that plays an important role in cell proliferation, tumorigenesis, and migration. Deficiency in *RASSF1A* leads to YAP activation and is a key factor in acquiring of malignant phenotypes, invasiveness, anti-apoptotic properties, and eventually transforming from bronchial epithelial cells into cancer cells (Dubois et al., 2016). Frequent promoter methylation of *APC* and *RASSF1A* found in 49% and 56% of patients, respectively. The detection of *P16* gene promoter methylation in serum or bronchoalveolar fluid/sputum has high specificity but low sensitivity. It may serve as a potential biomarker for NSCLC diagnosis but is not suitable for mass screening due to its low sensitivity (Tuo et al., 2018). However, the combined detection of *P16* and *RASSF1A* may be an effective biomarker for the early diagnosis of LC (Han et al., 2016). In a study by Feng et al. (2020) involving 89 Chinese NSCLC patients and 9 non-tumor patients, methylation of *SHOX2* (short stature homeobox 2) was significantly associated with NSCLC (*p* = 0.003 < 0.05) when using methylation-specific PCR (MSP). MSP has the advantages of rapidity, simplicity, specificity, and low cost, making it suitable for large-scale early screening of LC. The combined detection of *SHOX2* and *RASSF1A* gene methylation is also considered an excellent method for the early screening and monitoring of LC due to its high sensitivity and specificity (Li et al., 2020).

### 3.3 Female reproductive system cancer

#### 3.3.1 Breast cancer (BC)

Breast cancer is the most common cancer in women and the leading cause of cancer death. According to the latest statistics in 2020, female breast cancer ranks first among common cancers worldwide and is the fifth leading cause of cancer-related deaths worldwide (Sung et al., 2021). Traditional diagnostic methods for BC include mammography, MRI, biopsy, and histological analysis of tumor tissue. However, the incidence of BC is increasing year by year, especially in developing countries where screening and awareness are lacking. Therefore, liquid biopsy based on blood samples has emerged as a valuable tool for the early detection of BC. Biomarkers may enhance early detection of BC and provide symptomatic treatment options, thereby reducing the risk of death. Epigenetic features are considered good biomarkers for early detection, prognosis, and targeted therapy of BC, with DNA methylation being a potential biomarker for its early detection. For example, the down-regulation of *PAQR3* (progestin and adipoQ receptor family member III) expression in BC tissues is significantly associated with abnormal methylation of gene promoters (Chen et al., 2016). Methylation of *APC* and *RARB* genes can be detected in serum samples from patients with early BC (Swellam et al., 2015). Most promoters such as *ALDH1A2*, *ALDH1L1*, *GSTP1*, *p16*, *ESR1* and *PITX2* have been studied regarding BC methylation markers focusing on the prognosis of operable patients with BC (Sheng et al., 2017; Zhang et al., 2022). Recent studies (Lønning et al., 2022; Al-Moghrabi et al., 2024) have shown that *BRCA1* epigenetic mutations increase the risk of triple-negative breast cancer (TNBC). Monitoring the DNA methylation status of *BRCA1* has great potential as a pre-screening tool for these cancer forms. However, there have been few studies on the early diagnosis of BC so far. Methylation markers for early diagnosis of BC in recent years are summarized in Table 4; Figure 5.

**TABLE 4:** Candidate biomarkers of methylation in early diagnosis of breast, ovarian and cervical cancer.

Cancer type	Gene	Sample type	Type of marker	References
BC	*BRCA1*	Plasma, Tissue	Predictive, Screening	Nassar et al. (2020), Ruscito et al. (2021), Lønning et al. (2022), Al-Moghrabi et al. (2024)
	*CAV2*	Plasma	Screening	Uehiro et al. (2016)
	*GSTP1*	Plasma, Tissue	Diagnostic	Gurioli et al. (2018), Beňačka et al. (2022)
	*RASSF1A*	Plasma, Tissue	Diagnostic	Tang et al. (2016), Beňačka et al. (2022)
	*Sat2*	Plasma	Predictive	Khakpour et al. (2015)
	*ZNF217*	Plasma	Predictive	Khakpour et al. (2015)
OC	*AGRN*	Plasma	Predictive	Marinelli et al. (2022)
	*BCAT1*	Plasma	Predictive	Marinelli et al. (2022)
	*BRCA1*	Plasma, Tissue	Predictive, Screening	Ruscito et al. (2014), Yadav et al. (2020), Lønning et al. (2022), Terp et al. (2023), Al-Moghrabi et al. (2024)
	*BRCA2*	Plasma	Predictive, Screening	Yadav et al. (2020)
	*CAPN2*	Plasma	Predictive	Marinelli et al. (2022)
	*CDH1*	Plasma, Tissue	Diagnostic	Dvorská et al. (2019)
	*CDKN2A*	Tissue	Predictive, Screening	Abou-Zeid et al. (2011)
	*CDO1*	Plasma	Predictive	Marinelli et al. (2022)
	*CELF2*	Plasma	Predictive	Marinelli et al. (2022)
	*FAIM2*	Plasma	Predictive	Marinelli et al. (2022)
	*GPRIN1*	Plasma	Predictive	Marinelli et al. (2022)
	*GYPC*	Plasma	Predictive	Marinelli et al. (2022)
	*HIC1*	Plasma, Tissue	Screening, Diagnostic	Singh et al. (2020)
	*HOXA9*	Plasma, Tissue	Screening, Diagnostic	Singh et al. (2020)
	*MGMT*	Plasma	Predictive, Screening	Al-Moghrabi et al. (2024)
	*OPCML*	Plasma	Diagnostic	Terp et al. (2023)
	*PAX1*	Plasma, Tissue	Screening, Diagnostic	Dvorská et al. (2019), Fang et al. (2019)
	*PTEN*	Plasma, Tissue	Diagnostic	Dvorská et al. (2019)
	*RASSF1A*	Plasma, Tissue	Diagnostic	Dvorská et al. (2019), Terp et al. (2023)
	*RIPPLY3*	Plasma	Predictive	Marinelli et al. (2022)
	*SIM2*	Plasma	Predictive	Marinelli et al. (2022)
	*SOX1*	Plasma, Tissue	Screening, Diagnostic	Singh et al. (2021)
	*SRC*	Plasma	Predictive	Marinelli et al. (2022)
CC	*CADM1*	Plasma, Tissue	Predictive	Mersakova et al. (2014), Rong et al. (2019)
	*DAPK1*	Tissue	Predictive	Xiong et al. (2014)
	*EPB41L3*	Tissue	Screening	Kong et al. (2020)
	*FKBP6*	Plasma, Urine	Predictive	Guerrero-Preston et al. (2016)
	*INTS1*	Plasma, Urine	Predictive	Guerrero-Preston et al. (2016)
	*JAM3*	Tissue	Screening	Kong et al. (2020)
	*MAL*	Tissue	Predictive	Mersakova et al. (2014)
	*MEG3*	Plasma, Tissue	Diagnostic	Zhang et al. (2017b)
	*PAX1*	Tissue	Screening	Nikolaidis et al. (2015)
	*SIM1*	Plasma, Tissue	Diagnostic	Kim et al. (2018)
	*ZNF516*	Plasma, Urine	Predictive	Guerrero-Preston et al. (2016)

**FIGURE 5:**
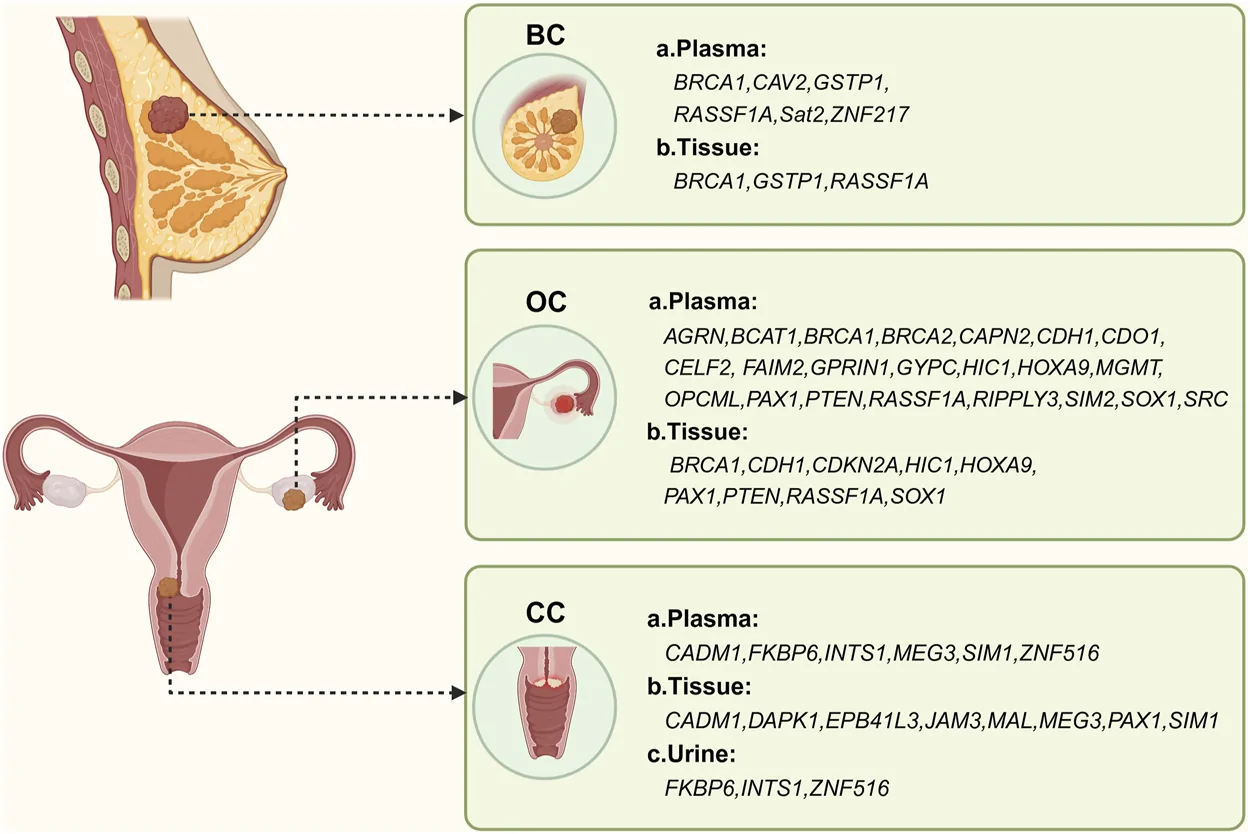
Methylation-based biomarker candidates in different samples for female reproductive system cancer diagnosis. Abbreviations: BC: a. Plasma: *BRCA1* (breast cancer 1), CAV2 (caveolin 2), GSTP1 (glutathione S-transferase pi 1), *RASSF1A* (Ras association domain family member 1), *Sat2* (spermine N1-acetyltransferase family member 2), *ZNF217* (zinc finger protein 217); b. Tissue: *BRCA1*, *GSTP1*, *RASSF1A*. OC: a. Plasma: AGRN (agrin), *BCAT1* (branched chain amino-acid transaminase 1), *BRCA1*, *BRCA2* (breast cancer 2), *CAPN2* (calpain 2), *CDH1* (cadherin 1), *CDO1* (cysteine dioxygenase type 1), *CELF2* (CUGBP, Elav-like family member 2), *FAIM2* (Fas apoptotic inhibitory molecule 2), *GPRIN1* (G protein regulated inducer of neurite outgrowth 1), *GYPC* (glycophorin C), *HIC1* (hypermethylated in cancer 1), *HOXA9* (homeobox A9), *MGMT* (O-6-methylguanine-DNA methyltransferase), *OPCML* (opioid binding protein/cell adhesion molecule-like), *PAX1* (paired box 1), *PTEN* (phosphatase and tensin homolog), *RASSF1A*, *RIPPLY3* (ripply transcriptional repressor 3), *SIM2* (single-minded family bHLH transcription factor 2), *SOX1* (sex determining region Y-box 1), *SRC* (SRC proto-oncogene, non-receptor tyrosine kinase); b. Tissue: *BRCA1*, *CDH1*, *CDKN2A* (cyclin-dependent kinase inhibitor 2A), *HIC1*, *HOXA9*, *PAX1*, *PTEN*, *RASSF1A*, *SOX1.* CC: a. Plasma: *CADM1* (cell adhesion molecule 1), *FKBP6* (FK506 binding protein 6), *INTS1* (integrator complex subunit 1), *MEG3* (maternally expressed 3), *SIM1* (single-minded family bHLH transcription factor 1), *ZNF516* (zinc finger protein 516); b. Tissue: *CADM1*, *DAPK1* (death-associated protein kinase 1), *EPB41L3* (erythrocyte membrane protein band 4.1-like 3), *JAM3* (junctional adhesion molecule 3), *MAL* (mal, T-cell differentiation protein), *MEG3* (maternally expressed 3), PAX1 (paired box 1), *SIM1*; c. Urine: *FKBP6* (FK506 binding protein 6), *INTS1* (integrator complex subunit 1), *ZNF516.* Figure created with BioRender.com.

#### 3.3.2 Ovarian cancer (OC)

According to the latest data from the American Cancer Society (SEER) in 2023, ovarian cancer is the most lethal gynecological malignancy, with a 5-year survival rate of 50.8%. High-grade serous ovarian cancer (HGSOC) accounts for 70% of ovarian cancer cases. Currently, the commonly used screening methods are clinical trials that combine the tumor markers CA-125 and transvaginal ultrasound. However, most patients do not exhibit specific symptoms in the early stages of OC and CA125 has limited sensitivity and specificity for early cancer detection (Gupta et al., 2019). The development of proteomics and genomics has led to the discovery of novel non-invasive methods for cancer detection and screening, such as tumor-associated autoantibodies and serum/plasma protein markers (Fortner et al., 2017). Unfortunately, most of these markers are only useful for advanced disease, so recent attention has focused on DNA methylation biomarkers for early screening of OC. DNA methylation is an early event in tumorigenesis and can be detected in ctDNA, making it a promising biomarker for early detection of OC (Liu et al., 2020). Data suggests that serum/plasma cfDNA methylation testing has a strong diagnostic accuracy for OC with a median accuracy of 85% (range 40–91%) (Guo et al., 2021). Several studies have identified candidate methylation biomarkers in plasma, such as *AGRN*, *BCAT1*, *CAPN2*, *CDO1*, *CELF2*, *FAIM2*, *GPRIN1*, *GYPC*, *RIPPLY3*, *SRC*, and *SIM2*, using sulfite sequencing and methylation-specific PCR. These biomarkers have shown high sensitivity and specificity for OC (Marinelli et al., 2022). Approximately 10%–20% of HGSOCs have *BRCA1* promoter methylation, which is an important risk factor (Lønning et al., 2022). MGMT methylation is also an important risk factor for HGSOCs (Al-Moghrabi et al., 2024). Dvorská et al. (2019) analyzed the regulation of *CDH1, PAX1, PTEN* and *RASSF1* tumor suppressor genes in benign and malignant ovarian tissues, as well as corresponding plasma samples, and demonstrated that the *CDH1* gene is a potential non-invasive diagnostic marker for OC. Methylation of the *CDH1* and *VIM* genes in epithelial ovarian cancer (EOC) can be detected using MS-HRM and is a reliable and sensitive method for detecting promoter methylation and early detection of EOC (Thakur et al., 2019). The MethyLight assay quantitatively analyzed the promoter methylation of *HIC1* and *HOXA9* in cfDNA from tissue samples, cancerous samples, and non-cancerous samples. This novel marker combination showed a sensitivity of 88.9% in serum cfDNA, making it suitable as a serum biomarker for early screening of OC (Singh et al., 2020). Studies have shown that *SOX1* can be used for indirect detection of serum cfDNA, along with *HIC1* and *HOXA9* multiplex detection (Singh et al., 2021), specifically in the northern Indian population. Research on the early diagnosis of OC is still ongoing, and recent years have seen a summary of OC methylation biomarkers, as shown in Table 4; Figure 5.

#### 3.3.3 Cervical cancer (CC)

Cervical cancer is ranked as the fourth most common cancer and the fourth leading cause of cancer death in women, with an estimated 6,04,000 new cases and 3,42,000 deaths worldwide in 2020 (Sung et al., 2021). Currently, routine screening for CC includes cytological tests, Pap tests, and HPV-DNA methods (Huh et al., 2015). Human papillomavirus (HPV) is a necessary but insufficient cause of CC, and preventive measures such as the HPV vaccine and screening can be taken. During the development of CC, various genetic and epigenetic changes occur in both the HPV and host genomes. After HPV gene methylation, HPV L1, HPVE6, and HPVE7 are present. E7 inactivates the retinoblastoma protein, while E6 inactivates p53. E6 can induce DNMT1 expression by inhibiting p53, and E7 can directly activate DNMT1 (Herbst et al., 2022). Methylation of gene promoters such as *CADM1*, *CCNA1*, *CDH1*, *DAPK1*, *FHIT*, *MAL*, *P16*, *PAX1*, *RAR-β*, and *RASSF1* can be considered as non-invasive biomarkers for early cervical lesions (El et al., 2021). The combination of plasma *CADM1* methylation and D-dimer testing is a biomarker that can be used for early screening of CC (Rong et al., 2019). Methylation of *EPB41L3* and *JAM3* is an accurate and feasible screening method for more severe lesions (CIN2) in CC tissues (Kong et al., 2020). A combination of promoter methylation tests for *FKBP6, INTS1,* and *ZNF516* can be detected early in blood cfDNA and urine (Guerrero-Preston et al., 2016). Abnormal methylation of *MEG3* (Zhang et al., 2017) and *SIM1* (Kim et al., 2018) may serve as potential plasma biomarkers for CC. In summary, eight methylation biomarkers for CC screening and early detection are described, while the others are listed in Table 4; Figure 5.

### 3.4 Urinary system cancer

#### 3.4.1 Bladder cancer (BCa)

Bladder cancer is the tenth most common cancer worldwide, with higher incidence in men than in women. It ranks as the sixth most common cancer and the ninth leading cause of cancer death in men (Sung et al., 2021). The most common malignancy of the urinary tract is BCa, and early detection is crucial for bladder preservation and improved survival. Cancer is caused by interactions between the environment and genetic and epigenetic factors, with DNA methylation being a common representation of epigenetics. Fluorescence *in situ* hybridization (FISH) is widely used for routine clinical detection of BCa, with a high sensitivity of 60%–80%. The utMeMA method, developed by Chen et al. (2020), is a rapid, high-throughput, noninvasive and promising method for early diagnosis of BCa. It can be used for detection of minimal residual tumor and assessment of DNA methylation in urine samples. Some studies have developed combined methylation assays for *HOXA9, PCDH17, POU4F2,* and *ONECUT2*, which are effective markers for early detection of BCa in urine samples from patients with hematuria (Wu et al., 2020). Another highly sensitive and specific noninvasive method for detecting primary BCa is the detection of methylated *NID2* and *TWIST1* genes in urine sediment using MSP (Renard et al., 2010). *SOCS1* (suppressor of cytokine signaling 1) protein is a negative regulator of the Janus kinase (JAK)/signal transducer and activator of transcription (STAT) signaling pathway and has tumor suppressor activity. In certain cancers, the methylation of CpG islands in the promoter region of the *SOCS1* gene inactivates the gene. The aberrant methylation of the CpG island in the promoter region of the *SOCS1* gene can be used as a biomarker for early prediction and diagnosis of BCa (Guan et al., 2020). Overall, there is limited research on methylation biomarkers for early screening and detection of BCa, as shown in Table 5; Figure 6.

**TABLE 5:** Candidate biomarkers of methylation in the early diagnosis of bladder and prostate cancer.

Cancer type	Gene	Sample type	Type of marker	References
BCa	*HOXA9*	Urine	Predictive, Diagnostic	Wu et al. (2020b)
	*NID2*	Urine	Predictive	Renard et al. (2010)
	*ONECUT2*	Urine	Predictive, Diagnostic	Wu et al. (2020b)
	*PCDH17*	Urine	Predictive, Diagnostic	Wu et al. (2020b)
	*POU4F2*	Urine	Predictive, Diagnostic	Wu et al. (2020b)
	*SOCS1*	Tissue	Predictive, Diagnostic	Guan et al. (2020)
	*TWIST1*	Urine	Predictive	Renard et al. (2010)
PCa	*GSTP1*	Plasma, Urine, Tissue	Predictive, Diagnostic	Ahmed, 2010; Zhou et al. (2019b)
	*PTGS2*	Urine	Diagnostic	Mikhaĭlenko et al. (2014)
	*RASSF1*	Tissue	Screening, Diagnostic	Ge et al. (2014), Skorodumova et al. (2016)

**FIGURE 6:**
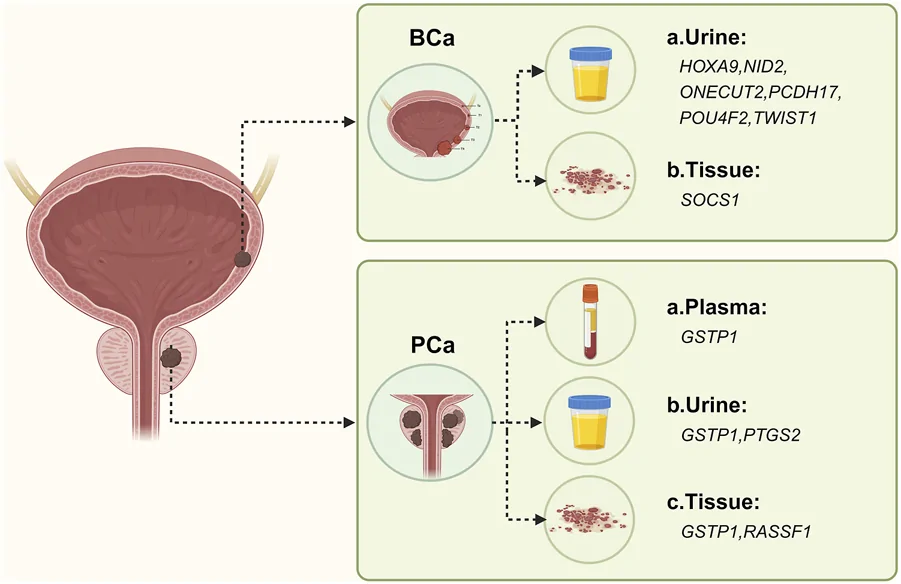
Methylation-based biomarker candidates in different samples for urologic cancer diagnosis. Abbreviations: BCa: a. Urine: *HOXA9* (homeobox A9), *NID2* (nidogen 2), *ONECUT2* (one cut homeobox 2), *PCDH17* (protocadherin 17), *POU4F2* (POU class 4 homeobox 2), *TWIST1* (twist family bHLH transcription factor 1); b. Tissue: *SOCS1* (suppressor of cytokine signaling 1). PCa: a. Plasma: *GSTP1* (glutathione S-transferase pi 1); b. Urine: *GSTP1, PTGS2* (prostaglandin-endoperoxide synthase 2); c. Tissue: *GSTP1, RASSF1* (Ras association domain family member 1). Figure created with BioRender.com.

#### 3.4.2 Prostate cancer (PCa)

According to statistics from the World Health Organization in 2020, prostate cancer is the fourth most common cancer worldwide and the second most common cancer and the fifth leading cause of cancer death in men in 2020 (Sung et al., 2021). The identified risk factors are limited to increasing age, family history of malignancy, certain genetic mutations (e.g., *BRCA1* and *BRCA2*) and disease (Lynch syndrome). Epigenetic mechanisms play an important role in the initiation and development of PCa, with gene-specific hypermethylation and hypomethylation being associated with PCa (Willard and Koochekpour, 2012). Currently, the standard diagnostic tools for PCa are prostate-specific antigen (PSA) serum tests and confirmation through histopathological examination. However, various biomarkers investigating epigenetic genetic alterations and their variants may be more predictive than PSA. The etiology of most cases of PCa is not fully understood, so there is a need to identify the molecular basis and early diagnostic markers for PCa. *GSTP1* (Glutathione S-transferase pi 1) expression is low in normal prostate luminal cells. However, in prostate intraepithelial neoplasia (PIN) and prostate adenocarcinoma (CaP), *GSTP1* is silenced due to cytosine-guanine phosphate (CpG) island promoter hypermethylation. Several studies have been conducted on PCa tissue samples, and they consistently demonstrate that *GSTP1* methylation is an early event in the carcinogenic process (Zelic et al., 2016). The most commonly used method for analyzing *GSTP1* methylation status in PCa research is methylation-specific polymerase chain reaction (MS-PCR). Other methods include bisulfite genome sequencing, pyrosequencing, methylation-specific dot blot assay, among others (Gurioli et al., 2018). *GSTP1* exhibits high specificity but low sensitivity as a biomarker for PCa. Combining *GSTP1* with traditional prostate-specific antigen (PSA) and other methylated genes can enhance early detection of PCa (Ye et al., 2023). In fact, a combination of three genes, *GSTP1*, *GAS6*, and *HAPLN3*, has been found to accurately classify and screen early PCa (Patel et al., 2019). Some meta-analyses suggest that *P16* methylation may serve as a potential biomarker for PCa (Feng et al., 2015). Although there have been few studies on methylation markers in PCa, *GSTP1* methylation remains the most extensively studied epigenetic marker in PCa. Other markers are listed in Table 5; Figure 6.

### 3.5 Other cancers

In 2020, the global incidence of thyroid cancer was 586,000, ranking ninth in terms of incidence, with a rate of 10.1/100,000 in women worldwide, which is three times higher than that in men (Sung et al., 2021). Thyroid cancer remains the most common endocrine malignancy, and its incidence has been gradually increasing in recent years. Sheikholeslami et al. (2021) noted that the methylation status of *DAPK1*, *P16*, *RASSF1*, *RUNX3*, *SLC5A8*, *TIMP3*, and *TSHR* promoters is a common and likely early event in lymph node metastasis and additional thyroid invasion. This information can be used for early diagnosis and differentiation of malignant and benign thyroid diseases.

Glioblastoma (GBM) accounts for the majority of gliomas (56.6%), making it the most common malignant primary brain tumor in adults with the worst prognosis (Ostrom et al., 2018). *MGMT* is potentially important for guiding the use of the chemotherapeutic agent temozolomide (TMZ) in GBM, and *MGMT* promoter methylation is significantly associated with improved overall survival in GBM patients (Sareen et al., 2022). *TP53* mutations can be detected in the early stages of 65% of secondary GBM multiforme (Rumpold et al., 2020), which provides a potential biomarker for early screening for extraneural metastases.

## 4 Conclusion

In conclusion, cancer epigenetics is prevalent in tumors of the digestive, respiratory, female reproductive, and urinary systems, and significant advancements have been made in this field. Epigenetic markers offer promising prospects for cancer screening and treatment. Tissue and free DNA-based epigenomics methods have been proposed for early detection, diagnosis, and treatment monitoring of various cancers, including colorectal cancer, lung cancer, breast cancer, bladder cancer, prostate cancer, and glioblastoma (Leal et al., 2020).

However, from a biological standpoint, despite substantial progress in gene methylation over the past decade, many unresolved questions persist. There is a need to develop optimized combinations of methylation markers for early tumor screening, and promising candidate methylation markers should be validated in prospective study cohorts and tested in large screening populations using high-quality studies.

Emerging liquid biopsy methods (free circulating nucleic acids, CTCs, and exosomes) have been utilized in recent years to discover, detect, and validate biomarkers. Data from studies conducted over the last 5 years demonstrate the feasibility of using epigenetic-based markers in body fluids and their potential for clinical translation. Therefore, there is an urgent need for large-scale, methodologically rigorous epidemiological studies to elucidate the potential role of methylation alterations in the development of various cancers and their impact on early detection in clinical samples such as blood, urine, sputum, feces, and tissues. With the rapid development of cfDNA analysis technology, liquid biopsy has a broader clinical application and prospect in oncology.

## Figures and Tables

**Table:** 

**CGI**	CpG islands
**5 mC**	5-methylcytosine
**GSTP1**	Glutathione-S-transferase P1
**EBF3**	Early B Cytokine 3
**CDMs**	Collagen Degradation Markers
**tCECs**	tumor-associated circulating endothelial cells
**HNSCC-CPs**	neck squamous cell carcinoma patients
**AA**	advanced adenomas
**CCA**	cholangiocarcinoma
**CFTR**	the cystic fibrosis transmembrane conductance regulator
**BSP**	Bisulfite Sequencing
**PYR**	Pyrosequencing Technology
**qPCR**	real-time PCR
**MS-HRM**	Methylation-Sensitive High-Resolution Melting
**MSRE**	Methylation-sensitive restriction enzyme
**MREBS**	Methylation-sensitive restriction enzyme bisulfite sequencing
**WGBS**	whole-genome bisulfite sequencing
**RRBS**	reduced representative bisulfite sequencing
**RCA**	rolling circle amplification
**COBRA**	Bisulfite restriction analysis
**BGS**	bisulfite genome sequencing
**MS-SnuPE**	Methylation sensitive single nucleotide primer extension
**NSCLC**	non-small cell lung cancer
**CRC**	colorectal cancer
**CEA**	Carcinoembryonic antigen
**ctDNA**	circulating tumor DNA
**CIMP-H**	the CpG island hypermethylation phenotype
**MethyQESD**	methylation quantitative endonuclease resistance DNA assay
**MSP**	methylation-specific quantitative PCR
**LTE-qMSP**	linear target enrichment quantitative methylation-specific real-time PCR
**ddPCR**	Droplet digital PCR
**MCTA-Seq**	Methylated CpG tandem amplification and sequencing
**HCC**	Hepatocellular carcinoma
**HBV**	hepatitis B virus
**HCV**	hepatitis C virus
**CTC**	circulating tumor cells
**AFP**	alpha-fetoprotein
**GC**	Gastric Cancer
**HP**	*Helicobacter pylori*
**PDA**	Pancreatic cancer
**BE**	Barrett‘s esophagus
**EAC**	esophageal adenocarcinoma
**LC**	Lung cancer
**LDCT**	low-dose computed tomography
**SCLC**	small cell lung cancer
**BALF**	bronchoalveolar lavage fluid
**BC**	Breast cancer
**OC**	ovarian cancer
**HGSOC**	high-grade serous ovarian cancer
**EOC**	early epithelial ovarian cancer
**CC**	Cervix cancer
**HPV**	Human papillomavirus
**BCa**	Bladder Cancer
**FISH**	Fluorescence *in situ* hybridization
**PCa**	Prostate cancer
**GBM**	Glioblastoma
**TMZ**	temozolomide
